# A framework for identifying factors controlling cyanobacterium *Microcystis flos‐aquae* blooms by coupled CCM–ECCM Bayesian networks

**DOI:** 10.1002/ece3.11475

**Published:** 2024-06-25

**Authors:** O. Tal, I. Ostrovsky, G. Gal

**Affiliations:** ^1^ Kinneret Limnological Laboratory Israel Oceanographic and Limnological Research Migdal Israel

**Keywords:** Bayesian network, causality, CCM, cyanoHAB, ECCM, ecosystem, Lake, *Microcystis*

## Abstract

Cyanobacterial blooms in freshwater sources are a global concern, and gaining insight into their causes is crucial for effective resource management and control. In this study, we present a novel computational framework for the causal analysis of cyanobacterial harmful algal blooms (cyanoHABs) in Lake Kinneret. Our framework integrates Convergent Cross Mapping (CCM) and Extended CCM (ECCM) causal networks with Bayesian Network (BN) models. The constructed CCM–ECCM causal networks and BN models unveil significant interactions among factors influencing cyanoHAB formation. These interactions have been validated by domain experts and supported by evidence from peer‐reviewed publications. Our findings suggest that *Microcystis flos‐aquae* levels are influenced not only by community structure but also by ammonium, phosphate, oxygen, and temperature levels in the weeks preceding bloom occurrences. We demonstrated a non‐parametric computational framework for causal analysis of a multivariate ecosystem. Our framework offers a more comprehensive understanding of the underlying mechanisms driving *M. flos‐aquae* blooms in Lake Kinneret. It captures complex interactions and provides an explainable prediction model. By considering causal relationships, temporal dynamics, and joint probabilities of environmental factors, the proposed framework enhances our understanding of cyanoHABs in Lake Kinneret.

## Graphical abstract

This study introduces a computational framework to analyze the causes of cyanobacterial harmful algal blooms (cyanoHABs) in Lake Kinneret. By integrating CCM and Extended CCM (ECCM) causal networks with Bayesian Network (BN) model, we uncovered significant interactions among various factors influencing cyanoHAB formation. The framework provides a more comprehensive understanding of the mechanisms driving cyanoHABs, capturing complex interactions and offering explainable prediction models by considering causal relationships, temporal dynamics, and joint probabilities of environmental factors.

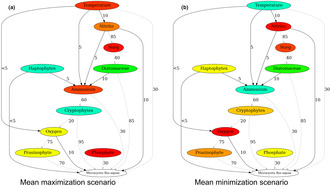

## 1 INTRODUCTION

Cyanobacterial harmful algal blooms (cyanoHABs) have a global impact, altering communities and producing toxins in lakes and water bodies. These events are influenced by climate change (Feng et al., 2016; Padisák, 1997; Paerl & Huisman, 2009) and various environmental factors (Benincà et al., 2008; Cao et al., 2006; Ninio et al., 2020; Reichwaldt & Ghadouani, 2012; Robarts & Zohary, 1987). CyanoHABs negatively impact their environment by altering their chemical and physical properties (Ibelings et al., 2003), and releasing toxins and allelopathic compounds (Sukenik et al., 2002). They also impact phytoplankton community structure and composition (Svirčev et al., 2014; Wang et al., 2020). Cyanobacterium *Microcystis flos‐aquae* is the dominant toxin‐producing species in Lake Kinneret and many other freshwater lakes and reservoirs (Harke et al., 2016). *Microcystis* blooms often occur in warm, nutrient‐rich waters with high levels of nitrogen and phosphorus. Additionally, *Microcystis* is known to thrive in alkaline conditions. The ability to cope with alkaline pH and to use different nitrogen species grants *Microcystis* the advantage over other phytoplankton species. Factors such as changes in water temperature, light, and water motions can also play a role in the development of *Microcystis* blooms (Wilhelm et al., 2020). Since 1995, Lake Kinneret has experienced significant and rapid ecological change, leading to increased frequency and magnitude of toxic blooms (Hadas et al., 2015; Markel et al., 2014).

The development of cyanoHABs prediction models has been studied and reviewed previously (Cruz et al., 2021; Rousso et al., 2020 and others). To date, cyanoHAB predictions have been carried out by both process‐based and data‐driven approaches (Rousso et al., 2020). However, it is important to understand the complex relations of cyanoHABs, community structure, and environmental factors.

The definition of causal relations between components of an ecosystem provides a valuable approach to understanding the key drivers and mechanisms behind specific events. By examining the relationships and interactions between the different components of an ecosystem, such as nutrient levels, phytoplankton communities, and environmental factors, we can identify the factors contributing to the development and persistence of cyanoHABs in Lake Kinneret. In recent years, causal relations between ecosystem components have been increasingly used to examine the drivers and impacts of the different components of ecological systems (Adams, 2003; Bonotto et al., 2022; Doi et al., 2021; Plowright et al., 2008; Sugihara et al., 2012). Traditionally, causal relationships between variables of the same system, assuming X and Y variables, are measured by the amount of information of past X that is encoded into future Y (Lucas, 2020; Moraffah et al., 2020; Zhao & Hastie, 2021). Granger Causality (GC) is used to identify and measure causality in time series (Granger, 1969). According to GC, X causes Y if the predictability of Y decreases when X is removed from the system. However, GC fails in dynamic systems consisting of variables that are not completely stochastic, with weak to moderate interactions.

An alternative method, Convergent Cross Mapping (CCM), was recently presented by Sugihara et al. (2012) CCM assumes (Deyle & Sugihara, 2011) that if two variables X and Y are of the same dynamic system, assuming X causes Y, then information about the state of X is embedded in Y and can be recovered. Interaction strength and directionality between the two variables can be quantified by measuring the prediction skill of the two variables by predicting an increasing number of X system states by an increasing number of Y system states until convergence of prediction skill. CCM also captures causal interactions that are not necessarily linear (Sugihara et al., 2012). This approach has been successfully implemented to reveal the causal effects in complex ecosystems (Barraquand et al., 2021; Chang et al., 2022; Nakayama et al., 2018; Zhang et al., 2022). Although CCM presents impressive performance in the identification of causal interactions in ecosystems, it carries essential drawbacks: (a) CCM does not supply information on synchrony between X and Y occurring by a strong driving force, (b) it does not specify whether the interaction is direct or indirect, and (c) CCM identifies causal interactions but does not supply information about their occurrence probabilities. The last point is extremely important for the understanding and possible management of complex and dynamic ecosystems. More recently, the Extended CCM (ECCM) presented by Ye et al. (2015) addresses the first two drawbacks. ECCM performs multiple CCM calculations at a range of time shifts of Y relative to X, to identify the lag of optimal prediction skill, which allows the identification of information flow direction. Bayesian networks (BN) is an approach that has been used to study causality in ecosystems (Aguilera et al., 2011; Barton et al., 2012; McCann et al., 2006) due to their probabilistic nature. BNs are probabilistic graphical models that use conditional probability distributions to specify the influence of the system's variables on a target variable (Milns et al., 2010). Yet, when the structure is learned from the data, it lacks directionality and is strongly affected by correlations. Therefore, the reliable construction of BNs requires the knowledge of domain experts.

Here, we suggest a novel causality analysis framework based on the use of CCM and ECCM for the construction of a target‐focused interaction network, on which the BN is calculated. Using the complex Lake Kinneret ecosystem as a case study, we constructed a computational framework to investigate the causes of toxic cyanobacterium *M. flos‐aquae* blooms. It is the first study in which BN models are constructed based on CCM and ECCM causal networks, and is applied here to the Lake Kinneret freshwater ecosystem as a case study in order to investigate the causes of the toxic *M. flos‐aquae* blooms.

## 2 METHODS

### 2.1 Study site and data

**FIGURE 1:**
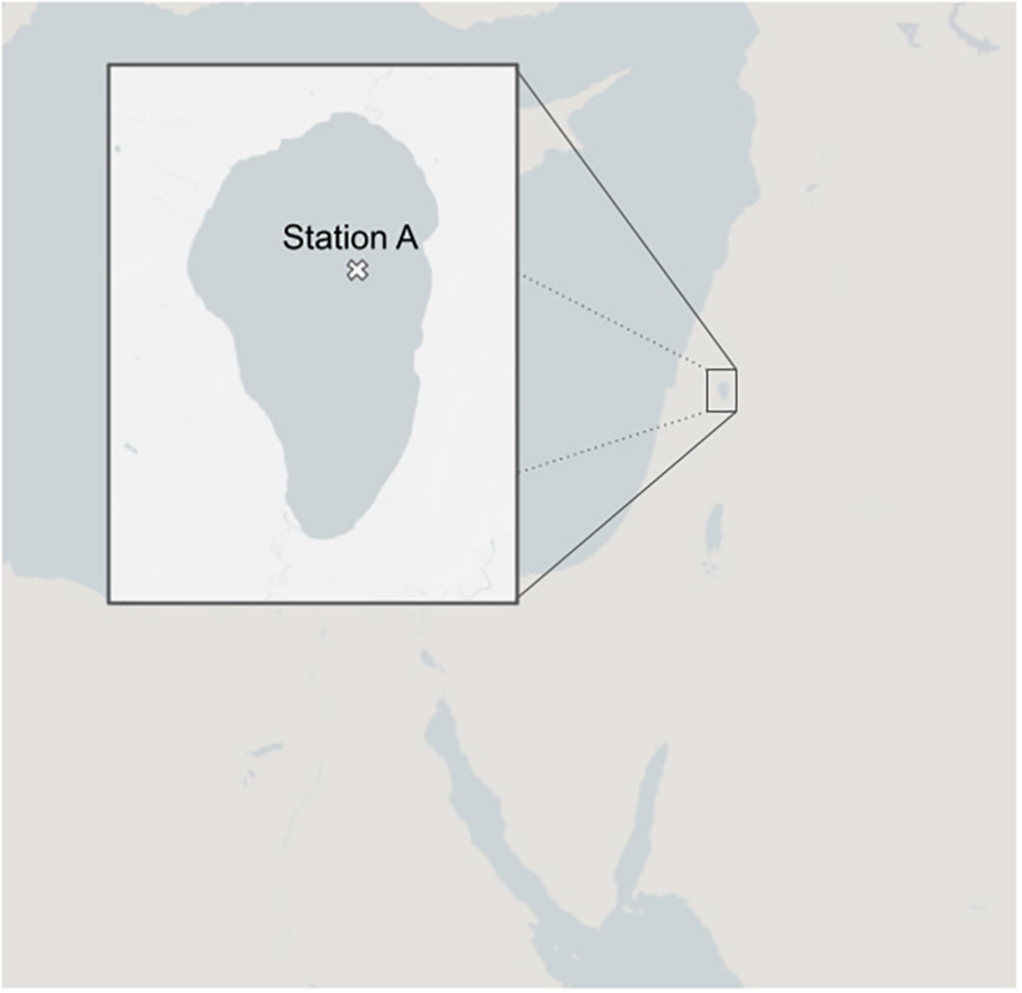
Study site. Lake Kinneret, northern Israel. The location of station A is above the deepest point of the lake (st. A coordinates: 32.82058, 35.58735).

Lake Kinneret (Sea of Galilee) is a 170 km^2^ warm meso‐eutrophic lake located in northern Israel (Figure 1). The lake has a maximum depth of about 43 m. CyanoHABs are especially critical in Lake Kinneret, the only freshwater lake in Israel and an essential source of drinking water, irrigation, fishing, and recreational activity. Understanding the dynamics leading to toxic blooms and producing accurate predictions of cyanoHABs would provide a powerful tool for proactive resource management and control of such events.

The Lake Kinneret Monitoring Program, which has been active since 1969, is conducted by the Kinneret Limnological Laboratory, Israel Oceanographic and Limnological Research. Routine measurements of physical, biological, and chemical variables are performed (Sukenik et al., 2014). The current study utilizes a 21 year dataset (2000–2020) consisting of measured phytoplankton biomass (prasinophyte, chlorophyta, diatomaceae, dinoflagellate, cyanobacteria, haptophytes, cryptophytes) in the water column and measurements of the main environmental components (nitrite, nitrate, ammonium, oxygen, particulate organic nitrogen, organic nitrogen, chloride, total dissolved phosphorus, dissolved organic nitrogen, phosphate, turbidity, and pH on a weekly‐biweekly basis), as well as surface water temperature and inflow volume. The variables were calculated as the sum per m (Paerl & Huisman, 2009) of the upper 10 m. All of the measurements used in this study are from the deepest station A, located at the center of the lake.

### 2.2 Data processing

The dataset was processed as follows. *Z*‐scores of 3 (three standard deviations from the mean) were considered as outliers and therefore discarded and interpolated. The dataset was resampled to a 5‐day resolution and was normalized to a range confined between 0 and 1 for the CCM calculations. For the BN approach, the data was categorized into three categories: ‘0’, ‘1’, and ‘2’ (Table S1).

### 2.3 Causal interactions

#### 2.3.1 Convergent cross mapping

CCM was utilized to elucidate the presence and direction of weak to moderate non‐linear causal interactions. The principle of CCM is based on the ability to predict the system state of a variable (X) by the system state of another variable (Y) (Deyle & Sugihara, 2011). If X is causal to Y, then information about X should be presented in Y, therefore the state space manifold reconstructed for Y should be able to predict the system's states of X (Sugihara et al., 2012). Here, the 21‐year time series was divided into multiple subsets by a 100‐week sliding window and a 10‐week gap between the windows. A time window of 100 time‐steps covers enough to capture at least one full annual ecological cycle, yet not be affected by trends or other long‐term effects. Moreover, selecting 100 time‐steps allows more robustness of the results by ensuring enough information for the CCM calculation. In case of a certain year in which the data consists of some months of lower data quality (such as missing measurements, malfunction sensor, etc.), yet data of higher quality is presented to the algorithm. While the optimal gap would be the use of a single time‐step that examines all possible subsets, it is computationally expensive. The selection of a 10 weeks gap reduces computational demands while maintaining an overlap of 90% so information loss is minor. The Augmented Dickey‐Fuller test was used to identify non‐stationarity in each of the subsets. In case a non‐stationary subset was identified, the difference in the subset was calculated.

Lagged coordinate vectors of the different variables were calculated, where E is an embedding dimension, and l is the lag step. The optimal E was selected based on the simplex projection (Sugihara & May, 1990), and the optimal l was selected from the first minimum in the mutual information between the time series and a shifted version of itself, using the Python package skccm (Nick Cortale, 2016). In addition, the S‐map method (Chang et al., 2021) was used to test the non‐linearity of the system with the PyEDM (Sugihara, n.d.) Python package, since in the non‐linear system, the prediction skill improves as lag increases. In case the optimal E or the optimal l were larger than 20, default values of *E* = 5 and *l* = 2 were set. We used this threshold in order to keep the information of the calculated system states in the scope of short‐ and mid‐term ecological effects.

The data was split into training (0.75) and test (0.25) subsets. Prediction skill (*p*) scores were calculated using an increasing number of system states (library size). Here, *p* was calculated as the mean *p* that was calculated from the sliding windows of converged cross mapping, for example, sliding windows of the same time series which were not of converged cross mapping were excluded.

A target‐focused network of interactions was constructed as follows: (a) *M. flos‐aquae* was set as the target; (b) CCM was calculated for all possible interactions of the target with all of the other variables; (c) the variables of causal interactions with the target were extracted; (d) CCM was calculated for all possible interactions within the causal variables that were extracted above; (e) a mean prediction skill (*p*) < 0.01 was used to filter out very weak interactions; (f) we considered an interaction if at least 10 of the sliding windows within a certain time series were of converged cross mapping and *p* ≥ 0.01. The initial CCM iterations were conducted to scan the dataset and to identify the most relevant variables for subsequent analysis. By performing these preliminary CCM calculations, we aimed to decrease the size of the dataset to include only the variables that exhibited causal interactions, thereby reducing computational requirements for subsequent ECCM analysis.

Surrogate time series were used to test the significance of CCM–ECCM results. Surrogate time series are created by modifying the data while preserving certain statistical properties of the original time series, such as its mean, variance, trend, and autocorrelation. If the result of the CCM calculation for the original time series was higher than the same calculation done on multiple surrogate time series, then it was considered significant. This means that the non‐linear correlation between the two time series is likely to be real and not due to chance. Here, Ebisuzaki's (PyEDM) method (Sugihara, n.d.) was used to generate surrogate datasets. If the observed prediction skill was greater than the 0.9 quantile of CCM prediction skill scores generated from 1000 surrogate time series, it was considered significant.

#### 2.3.2 Extended CCM

ECCM allows the detection of the optimal delay‐lag and discriminates the real unidirectional causal relationship from bidirectional causation by adjusting the cross‐map lag time (*l*) (Ye et al., 2015). This method is capable of identifying synchronization effects and false interactions that decrease CCM performance. In this method, CCM is calculated from a series of shifted datasets (Ye et al., 2015). In real causal interactions, the driving variable X can affect only the present or future Y. Therefore, Y can only predict the present or past values of X, but not its future values. Hence, the time lag between effect and cause must be non‐positive. If the optimal prediction skill lag of both “X causes Y” and “Y causes X” is equal to 0 and of similar magnitude, it means that both respond instantaneously to a strong driving force. If the optimal prediction skill of “X causes Y” is of a negative lag, and the optimal prediction skill “Y causes X” is of a positive lag, then the influence of X is strong enough to “enslave” Y due to synchrony (Ye et al., 2015). When both “X causes Y” and “Y causes X” present optimal prediction skill of negative lag, the causal interaction is bi‐directional. Given these guidelines, it is possible to validate the direction of causal interactions, determine the delay between the cause and the effect, and identify synchrony. Here, ECCM was tested for all causal interactions detected by the CCM analysis. For this analysis, a 400 data‐points frame was considered using *E* = 5, *l* = 2, maximum library size of 200, and shift range of −20 to 20 weeks. This information was used to refine and filter the interactions network.

### 2.4 Simulations

To validate the framework presented here, and to understand the limitations of CCM and ECCM, a series of experiments were conducted based on a well‐studied simulation of four species interactions adopted from equation (3) in Ye et al. (2015) (Figure S1a):

The simulated (eco)system consists of four species/factors whose direct time‐dependent relationships can be expressed by the following synthetic relationships that occurred at time steps (lag) of (*t* + 1):

$$\text{Y1}\left(t+1\right)=\text{Y1}\left(t\right)\left[3.9–3.\text{9Y1}\left(t\right)\right],$$

$$\text{Y2}\left(t+1\right)=\text{Y2}\left(t\right)\left[3.6–0.\text{4Y1}\left(t\right)–3.\text{6Y2}\left(t\right)\right],$$

$$\text{Y3}\left(t+1\right)=\text{Y3}\left(t\right)\left[3.6–0.\text{4Y2}\left(t\right)–3.\text{6Y3}\left(t\right)\right],$$

$$\text{Y4}\left(t+1\right)=\text{Y4}\left(t\right)\left[3.8–0.35\text{Y3}\left(t\right)–3.\text{8Y4}\left(t\right)\right],$$

These equations simulate self‐dynamics together with direct dependence of Y2 on Y1, Y3 on Y2, and Y4 on Y3, and consist of complex direct and indirect interactions (Figure S1a). Although the network is small, it implies similar challenges associated with the analysis of real‐world ecological interactions. We are aware of the fact that the simulated system was homogeneous in manners of system stability and influence of external drivers, while their influence in real‐world systems fluctuates over time. Therefore, heterogeneous data were simulated by generating a weak causally connected dataset by multiplying random components by Y2 and Y3, weakening the interactions of Y1, Y2, and Y3 (Figure S1b). The strong and the weak causally connected data were concatenated (1/3 strong causal interactions, and 2/3 weakened causal interactions) into a single heterogeneous dataset. CCM was analyzed for the two synthetic types of data analyses—with and without implementing sliding windows.

### 2.5 Bayesian network

The Python package bnlearn (Taskesen, 2020) was used to construct a target‐oriented BN model based on categorized (Table S3) 7‐year historical data (2014–2020) (Figure S3). BNs are the non‐parametric statistical method that describes the Bayesian probabilities of the system's components by directed acyclic graphs (DAG). Typically, the construction of BN involves multiple steps detailed in Chen and Pollino (2012) and Marcot (2017). In our study, BN inference was used to elucidate the conditions that may promote the maximization or minimization of *M. flos‐aquae* blooms. The BN was constructed based on causal interactions identified by CCM and ECCM. Here, the time series were shifted according to the lag of the maximum *p*, aligning the cause and the effect that were presented to the BN model. Since BN cannot represent feedback loops, it has to be calculated on a DAG structure. The CCM interactions network was processed as follows: (a) feedback loops were identified using the Python package Networkx (Hagberg et al., 2008); (b) feedback loops were removed by identifying a feedback loop, and truncating the loop after the target node, or before a confounder node; and (c) sink nodes (nodes which do not consist of out‐edges and are not *M. flos‐aquae*) were removed. The conditional probability tables were calculated from the categorized dataset. Quantiles were utilized as discretization thresholds, although the use of ecological thresholds might be more accurate and intuitive to interpret. This was made due to the challenge of identifying specific thresholds for each variable and ensuring adequate sample sizes of the different categories. We used the 0.75 quantile of the *M. flos‐aquae* biomass concentrations as a threshold.

The structure of the BN model was initially constructed based on the results obtained from the CCM calculations. CCM identified causal relationships and dependencies between variables based on the time series data. The network structure that was inferred from CCM reflects the temporal dynamics and causal relationships present in the original dataset. After constructing the structure of the BN model, using CCM results, parameter learning was performed using the categorized data. Then, conditional probability distributions (CPDs) were learned from the categorized data. Categorization reduced the complexity of the data by categorizing continuous variables into categories, therefore simplifying the relationships between variables. Using different categorization cutoffs changed the CPDs. Therefore, categorization cutoffs were carefully selected. BN was also constructed by a structure learning approach directly from the categorized datasets using the HillClimbing algorithm (Marcot, 2017). Structure learning aims to uncover the network's architecture, while parameter learning estimates the conditional probabilities given a fixed structure.

### 2.6 Sensitivity analysis

Sensitivity analysis was used to assess the impact of changes in the input parameters of the BN model on the output of the model. It allowed us to understand the robustness of the model and to identify the input parameters that have the greatest impact on the output of the model. We constructed up to 20,000 random permutations of different environmental scenarios, which were used as input vectors to the BN model. Then, the BN model's inputs and outputs were used for the estimation of model sensitivity. We analyzed the sensitivity of the BN model to each of the variables by calculating the mean contribution of each variable (mean residuals). The sum of the means absolute values of both *Microcystis* bloom maximization and minimization was considered as the variable's contribution.

### 2.7 Computation

**FIGURE 2:**
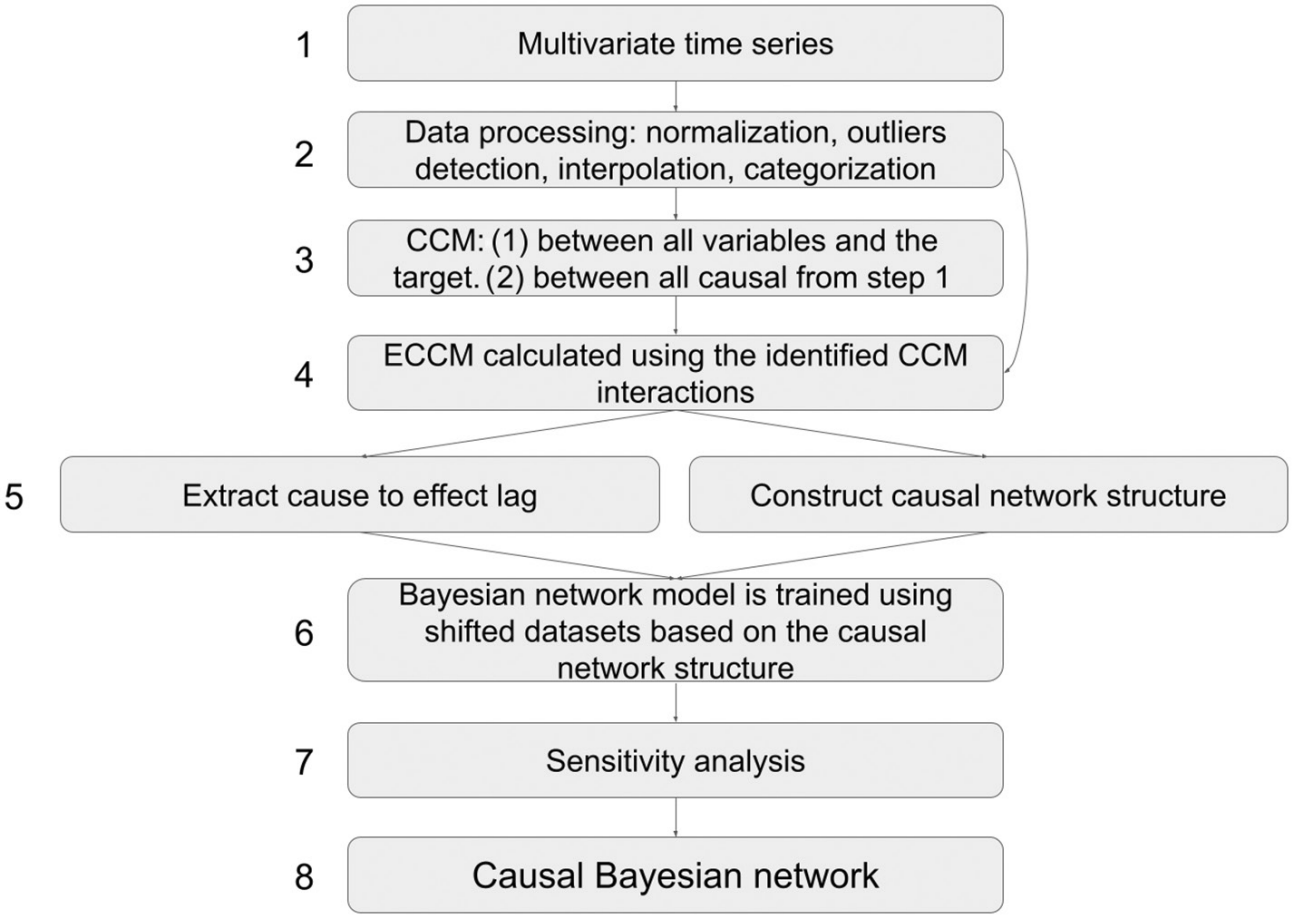
Schematic description of the framework implemented in this study. (1) Multivariate time series consists of the target (*Microcystis flos‐aquae*) and the other variables; (2) data processing, including normalization, outliers detection, interpolation, and categorization; (3) CCM is calculated between the target(s) and all of the other variables, followed by a second CCM step between all the causal variables; (4) causal interactions from step 3 are validated and further filtered by ECCM analysis; (5) cause‐to‐effect lags extractions, and construction of the causal network structure; (6) parameters are shifted according to cause‐to‐effect lags, and used for BN model training, based on network structure from step 5; (7) model sensitivity evaluation based on 20,000 permutations; and (8) causal Bayesian network model.

All calculations, analysis, and visualization were carried out under the Python environment and the relevant packages as described above. A schematic illustration of the process is presented in Figure 2.

## 3 RESULTS

### 3.1 Blooming patterns of *Microcystis flos‐aquae*

**FIGURE 3:**
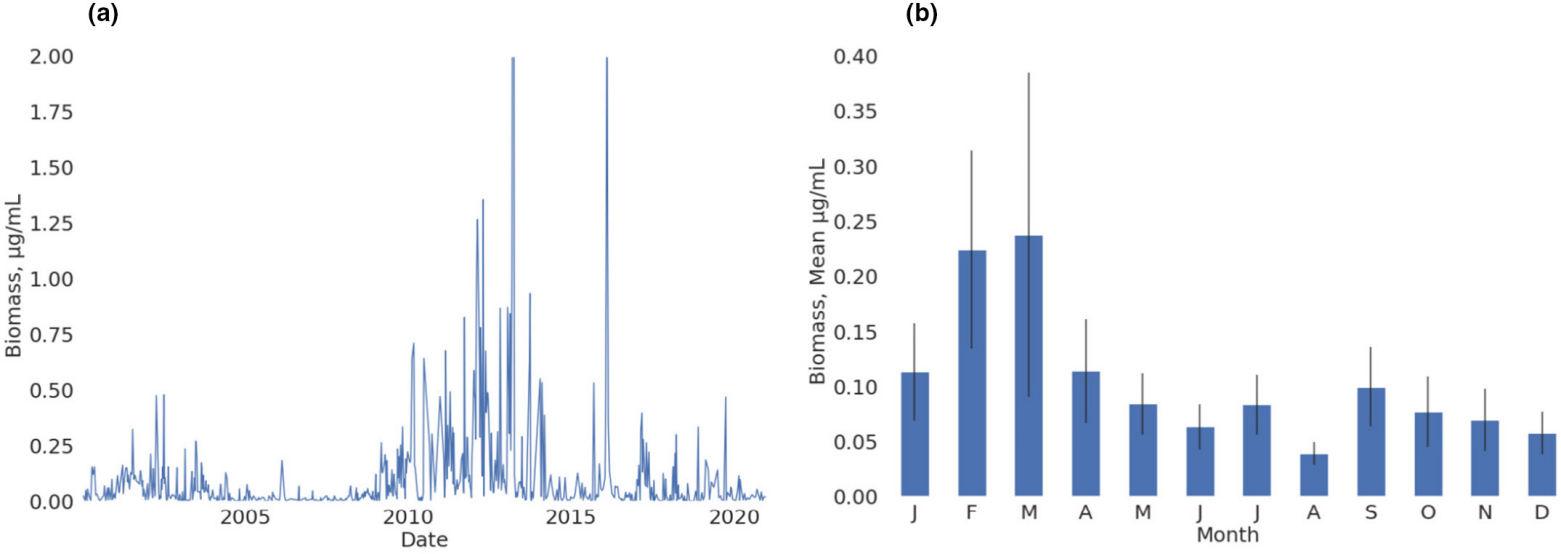
(a) *Microcystis flos‐aquae* values between years 2000 and 2020; and (b) monthly means.

Cyanobacterium *M. flos‐aquae* has been observed in Lake Kinneret from the beginning of lake monitoring (1969), but only since 1996 it has frequently formed distinct winter–spring blooms (Figure 3a). During these blooms, the peak biomasses were moderate before 2009; then, between 2010 and 2016, higher peak biomasses were detected; since then, only irregular blooms have taken place. Annual dynamics observed during the last 21‐year period show that *M. flos‐aquae* abundance starts to increase in January, reaching the peak values usually during the second half of February to beginning of March. The minimal biomass is detected in August, with a following small increase in September (Figure 3b).

### 3.2 Causal interactions

#### 3.2.1 Method validation using synthetic time‐series

**FIGURE 4:**
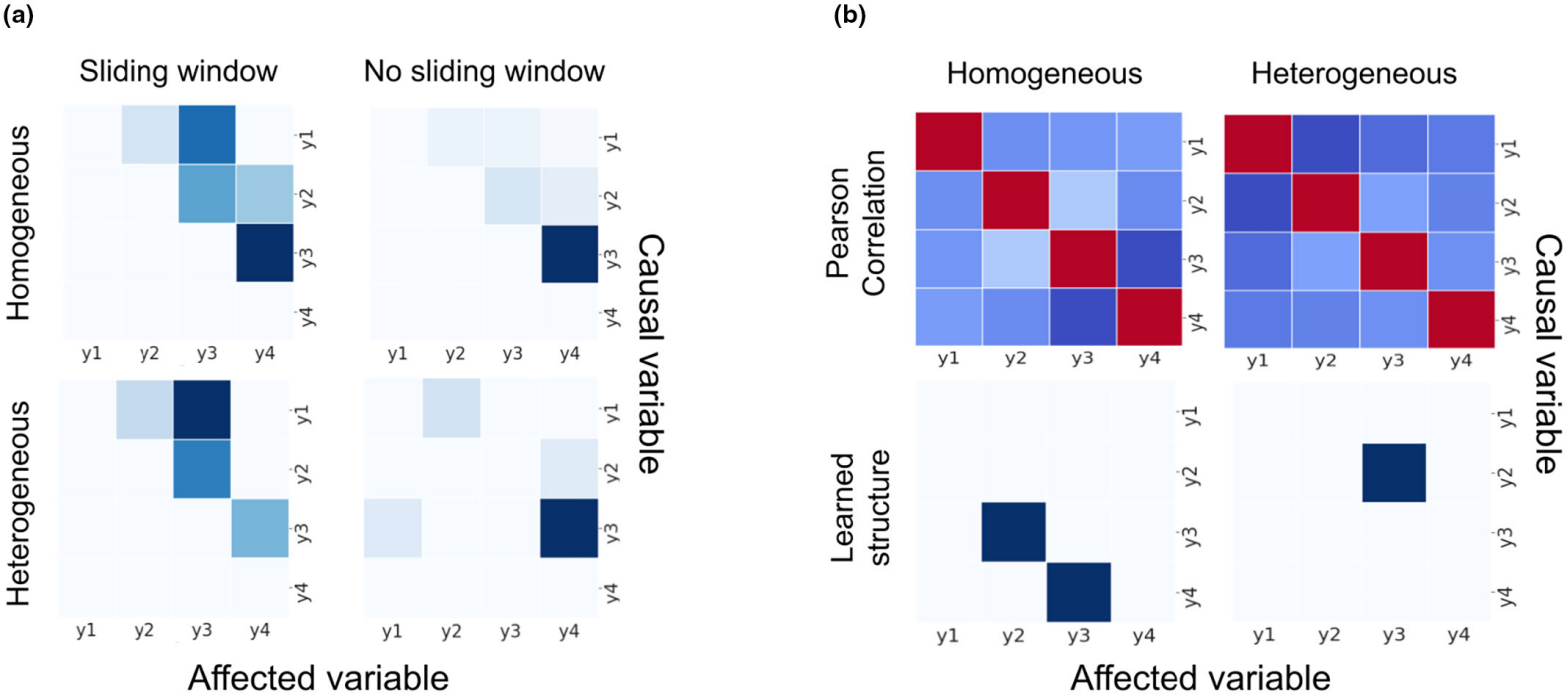
The results of the proposed framework that were calculated using a simulation adopted from Ye et al. (2015). The simulated system consists of four factors y1, y2, y3, and y4 with the relationships described in Figure S1. (a) Homogeneous and heterogeneous datasets CCM were calculated using a sliding‐window and without a sliding‐window (color scale shows CCM prediction skill. Light blue‐weaker, Dark blue‐stronger); and (b) pearson correlations (color scale shows correlation score. Blue‐negative. White‐zero. Red‐positive) and Bayesian structure learning results suggest different interactions; In both subplots the *x*‐axis is the affected variables, *y*‐axis is the causal variables.

The CCM–ECCM approach implementation was validated using a well‐studied synthetic dataset of four components consisting of direct and indirect interactions (Figure S1). CCM calculations of the simulated homogeneous time series, without sliding windows, successfully reconstructed the results presented by Ye et al. (2015) (Figure 4a). In addition to the homogeneous dataset, another version was created, in which the strong causal relations were weakened during the last two‐thirds of the simulation, better representing the dynamics in a real ecosystem. The sliding window approach identified more causal interactions compared to the single‐frame approach. The utilization of sliding windows reduced the identification of false interactions, which occurred when a single frame was used. The CCM calculation of the homogeneous time series using a sliding window technique identified the three direct interactions (y1 → y2, y2 → y3, and y3 → y4), however, missed a single indirect interaction of y1 → y4. These results are similar to the homogeneous without sliding‐window simulation, however produced higher CCM values, despite the single indirect y1 → y4 interaction. CCM calculation of the heterogeneous time series, without sliding windows, identified two direct interactions (y1 → y2 and y3 → y4), a single indirect interaction (y2 → y4) which was not identified by the sliding window approach, and a single false interaction. Using sliding windows, CCM of the heterogeneous time series identified the three direct interactions but missed two indirect interactions (y1 → y4, and y2 → y4).

We compared the proposed CCM–ECCM approach to structure learning and Pearson correlations (Figure 4b). Structure learning failed to identify the simulated causal interactions, two false interactions in the homogeneous time series, and a single true direct interaction (y2 → y3) in the heterogeneous dataset (Figure 4b). Pearson results were too noisy (as demonstrated by the complete lack of zero correlation scores in Figure 4b) and lacked information regarding directionality (by definition and demonstrated by the symmetry of correlation scores in Figure 4b).

#### 3.2.2 Identification of causal interactions in Lake Kinneret historical records using coupled CCM–ECCM

**FIGURE 5:**
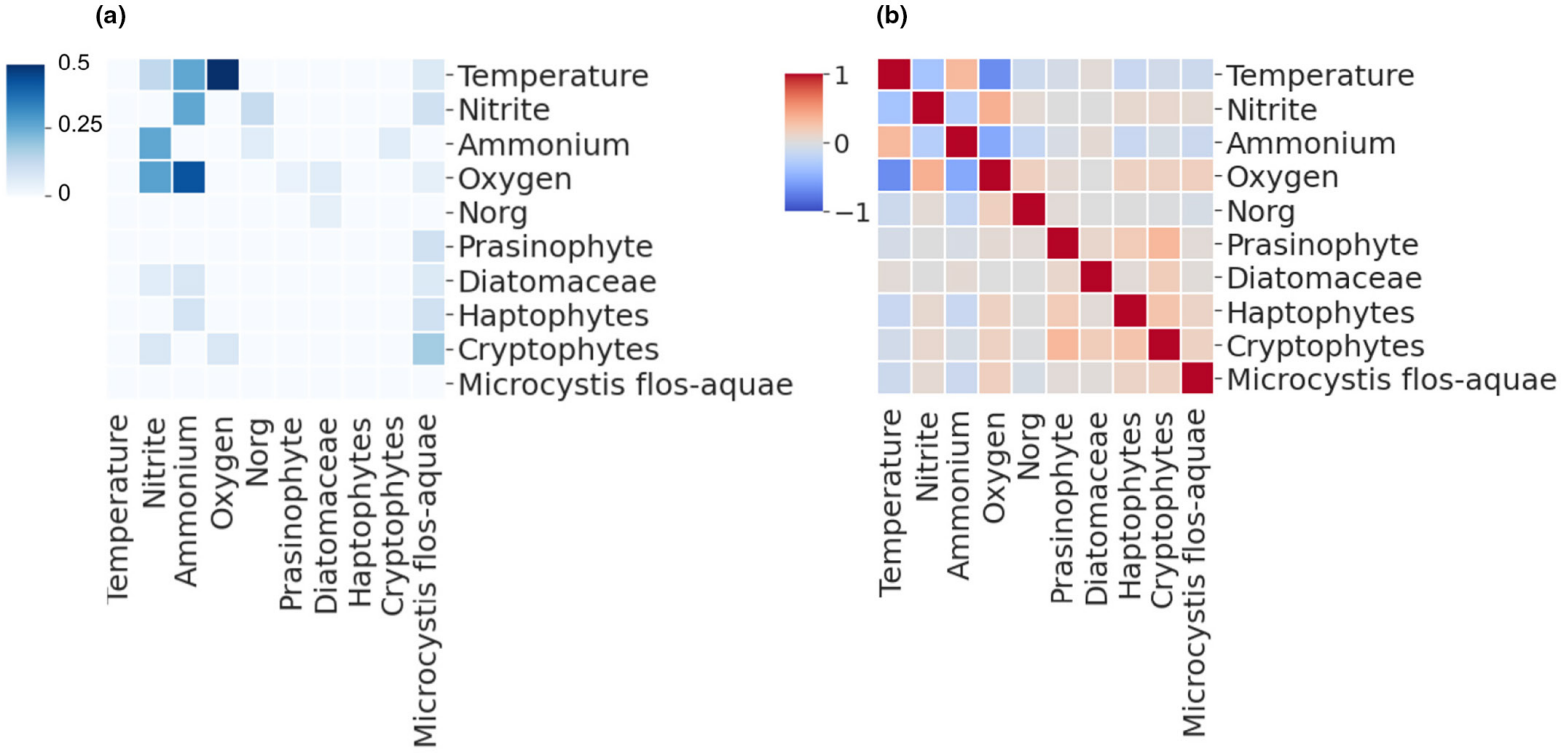
(a) CCM and ECCM interactions were calculated from historical records from the years 2000–2020. The color scale shows the CCM score in the range of 0 (light blue) to 0.5 (dark blue). The *x*‐axis is the affected variables, *y*‐axis is the causal variables. (b) Correlations between the same causal variables (color scale shows Pearson correlations between −1 [blue] and 1 [red]).

CCM results presented a complex array of interactions between the environmental (physical and chemical) variables and phytoplankton components (Table S2). The chemical parameters affect biological parameters that in turn affect chemical parameters. For example, nitrite → N (organic) → diatomaceae → ammonium → N (organic) (Figure 5a). This kind of feedback loops has to be simplified by truncation into DAG. Although more complex models can be more accurate, they may be more challenging to understand and interpret. In addition, too many nodes in a BN model can have a number of negative effects, including reduced accuracy, increased computational complexity, and overfitting (Marcot, 2017). Therefore, due to the complex network involving phytoplankton, individual species were aggregated by their taxonomic groups.

Some of the causal interactions that were revealed by CCM (Figure 5a) are correlated (Figure 5b), while the other interactions correlate weakly or do not correlate at all. If the framework identifies causal relationships between variables that are not correlated according to Pearson correlation scores, it suggests: (a) the presence of indirect or non‐linear causal relationships; (b) the presence of strong force affecting both variables; or (c) interactions of the opposite direction. This highlights the importance of considering causal relationships beyond simple correlations, especially in complex systems where indirect or non‐linear effects may play a significant role. For example, correlation cannot distinguish between “oxygen → temperature” and “temperature → oxygen” (Figure 5b).

ECCM was used to calculate time‐delayed interactions and to identify synchrony and false discoveries in CCM results. A total of 23 pairs, which represented all the possible interactions between all the environmental and biological variables and *M. flos‐aquae*, were examined by CCM in the first iteration. Of those, nine variables (Figure 5) were of CCM prediction skill above 0.01 and used in the second iteration. All possible interactions (71, excluding self‐interactions) between these variables were calculated in the second iteration. A total of 55 interactions presented converged prediction skill above or equal to 0.01. ECCM was calculated for these 55 pairs, and 28 were found as true interactions. These pairs were validated using 1000 surrogates for each interaction (Figure S3). We examined multiple surrogate cutoffs (0.9, 0.95, and 0.975) (Table S3), and found that the BN model of the interactions above the 0.95 quantiles is of higher accuracy compared to the other cutoffs (Table S2). Although it was of higher accuracy (0.876) in comparison to the accuracy of the 0.9 quantile cutoffs model (0.841), it lacked the interaction “oxygen → *M. flos‐aquae*,” which was present in the 0.9 quantile cutoffs. The 0.9 quantile interactions also consisted of interactions supported by domain experts and previous research, which are discussed further in the discussion section. Therefore, we used the 0.9 cutoffs.

**FIGURE 6:**
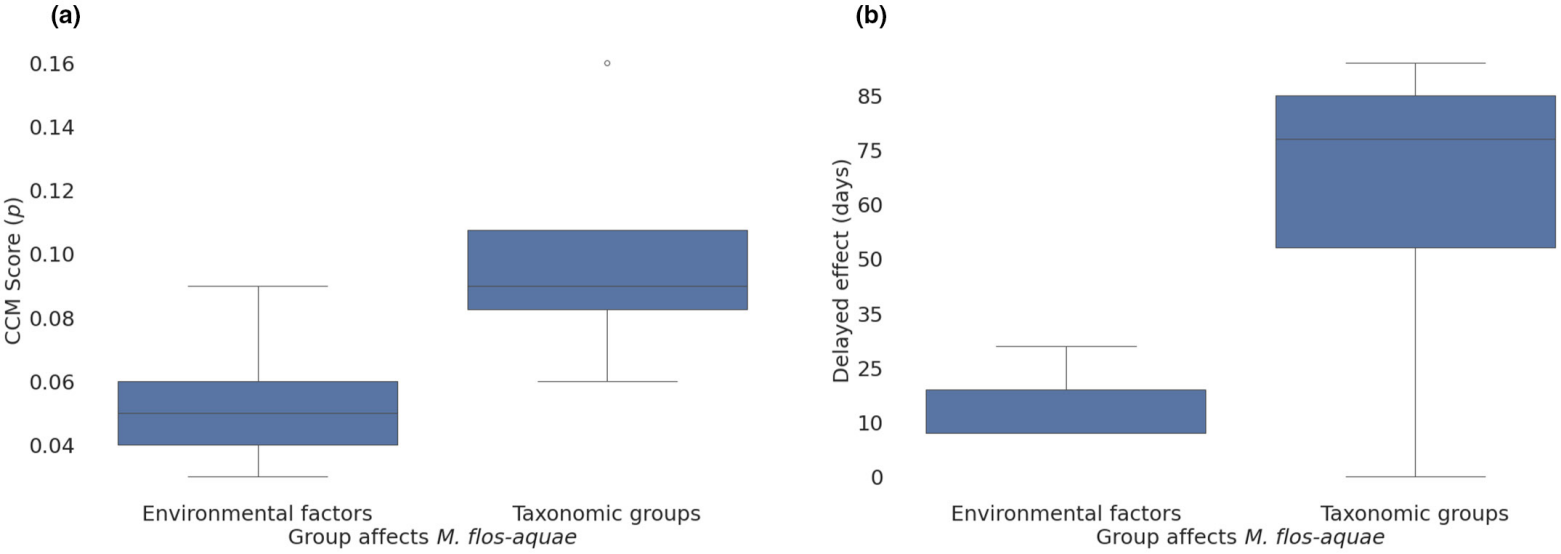
The environmental and biological interactions with *Microcystis flos‐aquae* are presented as boxplots of (a) CCM prediction skill scores, and (b) delayed effect values calculated from ECCM results. The boxplots are categorized to biological (*n* = 4) and environmental (*n* = 5) components.

Interestingly, the lower cutoffs consisted of more interactions involving biological variables (Figure S3). Only four interactions involved biological variables above the 0.975 cutoff (“diatomaceae → ammonium,” “temperature → *M. flos‐aquae*,” “cryptophytes → *M. flos‐aquae*,” and “nitrite → *M. flos‐aquae*”), while eight of the environmental variables interactions are above this cutoff, especially interactions involving only environmental variables, for example, “temperature → oxygen” or “oxygen → nitrite.” Although the interactions involving environmental variables were of higher CCM scores, their effect on *M. flos‐aquae* is weaker (Figure 6a). The biological variables that affect *M. flos‐aquae* were of longer term (Figure 6b) compared to the environmental variables, and the effect of environmental variables on biological variables was of longer lag (Table S2).

A total of 9 variables constructed the final network. The causal interactions were also examined by ECCM, which identified and discarded six false interactions. Of the 28 interactions, using the interactions above the 0.9 quantile, 25 (89.2%) interactions were above this threshold and considered significant (Figure S3).

The CCM scores of the phytoplankton parameters affecting *M. flos‐aquae* were higher (median = 0.09, mean = 0.1, std = 0.042) in comparison to the scores of the environmental parameters affecting *M. flos‐aquae* (median = 0.05, mean = 0.054, std = 0.023) (Figure 6a). The median lag (days) between the cause to the effect of the environmental parameters (median = 10, mean = 16.5, std = 11.5) was of shorter lag in comparison to the interactions of the biological parameters (median = 77.5, mean = 62.5, std = 42.9) (Figure 6b).

**FIGURE 7:**
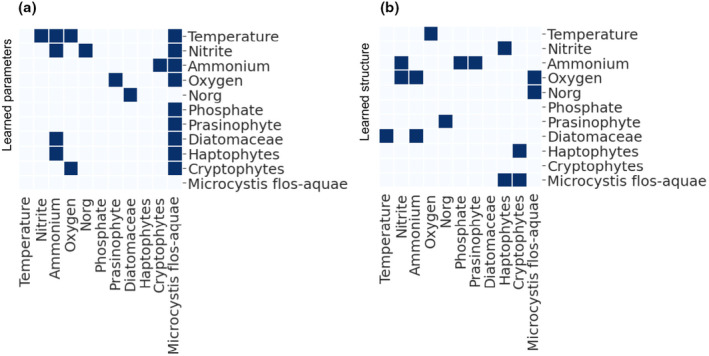
Networks of causal interactions that were identified by the proposed framework compared to network structure identified by structure learning approach. (a) CCM–ECCM approach, feedback interactions were removed, and (b) structure learning. *x*‐axis is the affected variables, *y*‐axis is the causal variables. The values are binary, indicating the presence (dark color) or absence (no color) of an interaction.

We did not identify direct interactions between ammonium or phosphate and *M. flos‐aquae*, but indirect effects were identified with ammonium, which is central in the constructed network (Figure 5a). Both ammonium and phosphate are known to affect *Microcystis* (Harke et al., 2016), therefore we added “ammonium causes *M. flos‐aquae*” and “*phosphate causes M. flos‐aquae*” (Imai et al., 2008) to the BN model (Figure 7a).

The structure learning approach in which the Bayesian network is calculated by the Hillclimbing algorithm, and the parameters learning approach, in which the parameters are calculated based on a given network structure, were compared using the historical dataset. Three interactions were found to overlap (Figure 7b): “temperature → oxygen,” “diatomaceae → ammonium,” and “oxygen → *Microcystis*.” The structure learning approach identified fewer interactions (15) than the CCM–ECCM structure learning (18 excluding ammonium and phosphate). Like the simulation results, Pearson correlations were too noisy and did not provide information regarding interaction directionality (Figure 5b).

### 3.3 Directed acyclic graph and Bayesian network model

**FIGURE 8:**
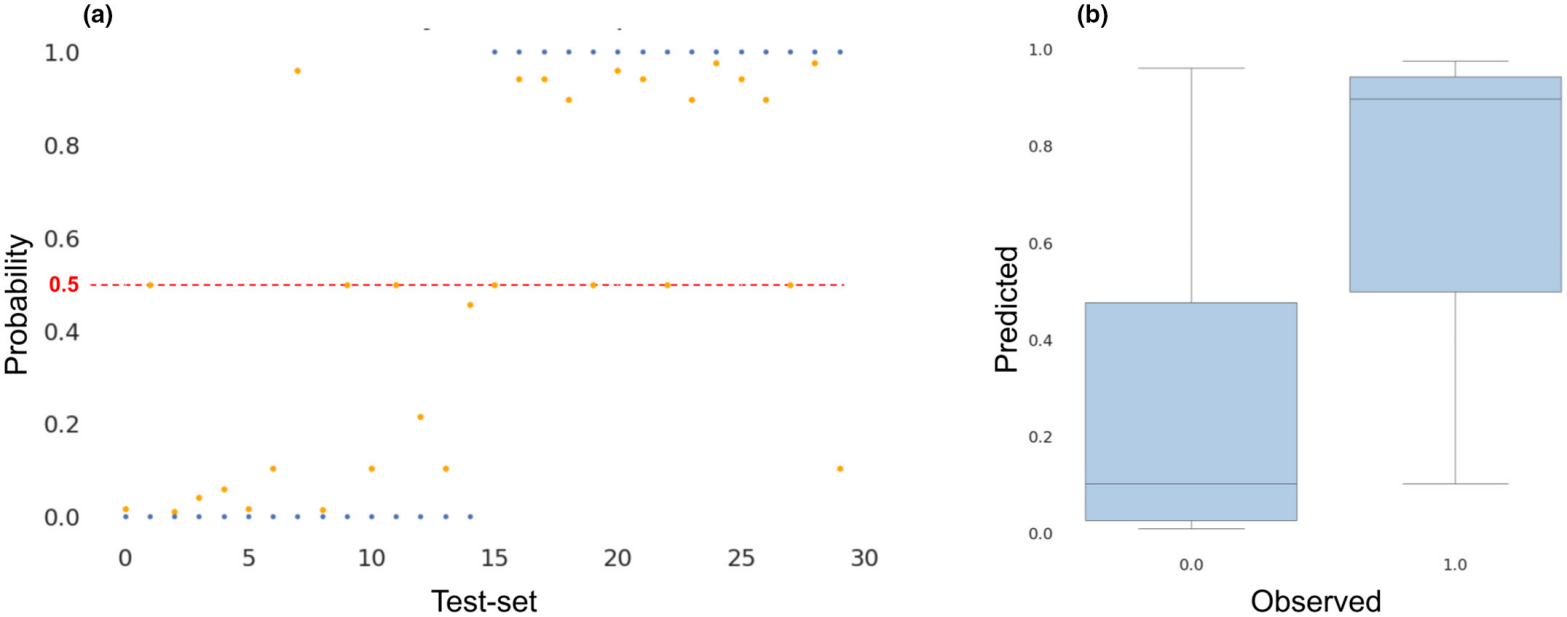
Model validation. Bayesian network model was trained using the training dataset, and validated using the testing dataset. Probability <.5 is considered ‘0’, and probability ≥.5 is considered ‘1’. (a) Observed (blue) and predicted probabilities (orange) of *Microcystis flos‐aquae* bloom formation. (b) Box plot of observed versus predicted probabilities of *M. flos‐aquae* bloom formation.

The causal interactions network was converted to a DAG in order to construct a BN. The resulting DAG consisted of 11 nodes and 20 interactions, including the two direct interactions that were added, “ammonium → *M. flos‐aquae*” and “*phosphate* → *M. flos‐aquae*.” To avoid over‐fitting, we confirmed that the number of cases of each state, for each variable, was >20 (Chen & Pollino, 2012). The dataset was split into training (0.75) and test (0.25) subsets. The BN model was calculated based on a shifted time series, where the cause and the effect were aligned according to the lags identified by ECCM analysis (Table S2). The model was evaluated based on a confusion matrix (Figure S4), accuracy, and AUC scores. *M. flos‐aquae* BN model (Figure 8a) achieved an accuracy of 0.841 and AUC score of 0.842 (Table S3) considering a probability cutoff larger than or equal to .5 (Figure 8b).

### 3.4 Sensitivity analysis and cyanobacteria blooming/non‐blooming scenarios

**FIGURE 9:**
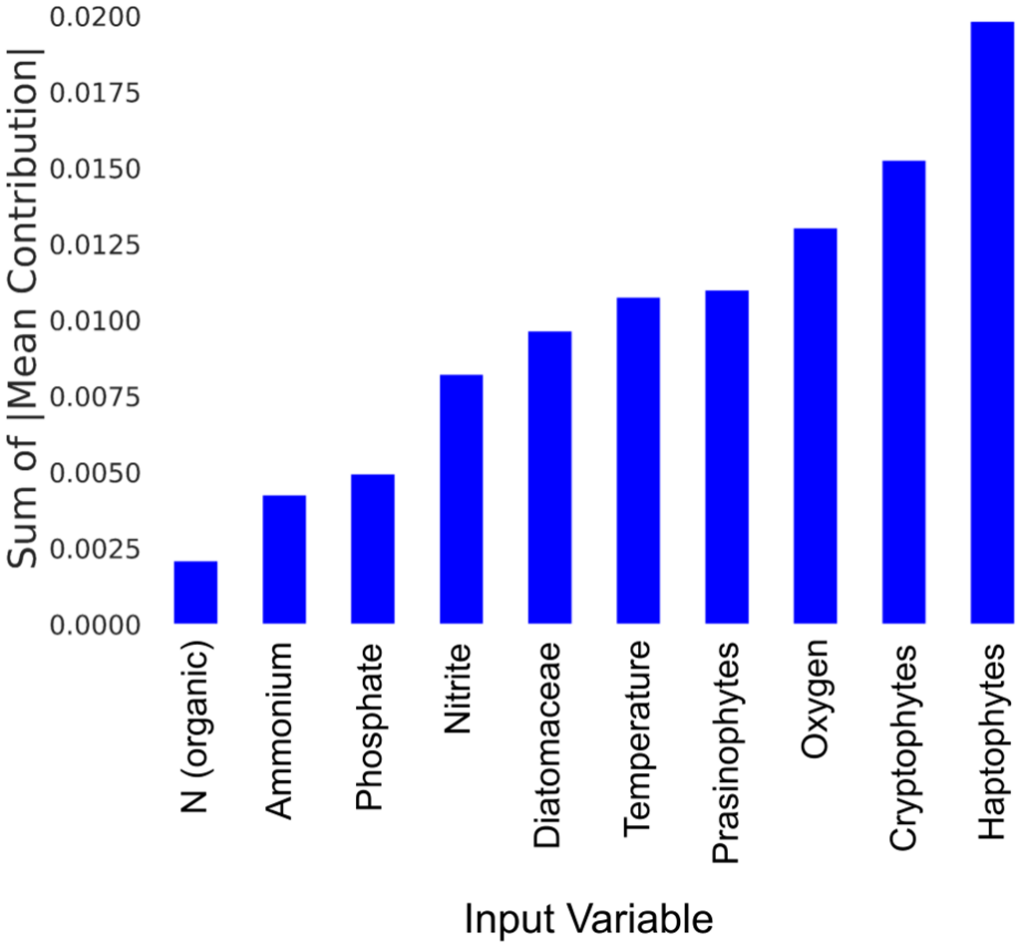
Sensitivity analysis of the BN model. The bar plot displays the sum of absolute values of the means of the different features to the model's output, which is the blooming probability of *Microcystis flos‐aquae*.

**FIGURE 10:**
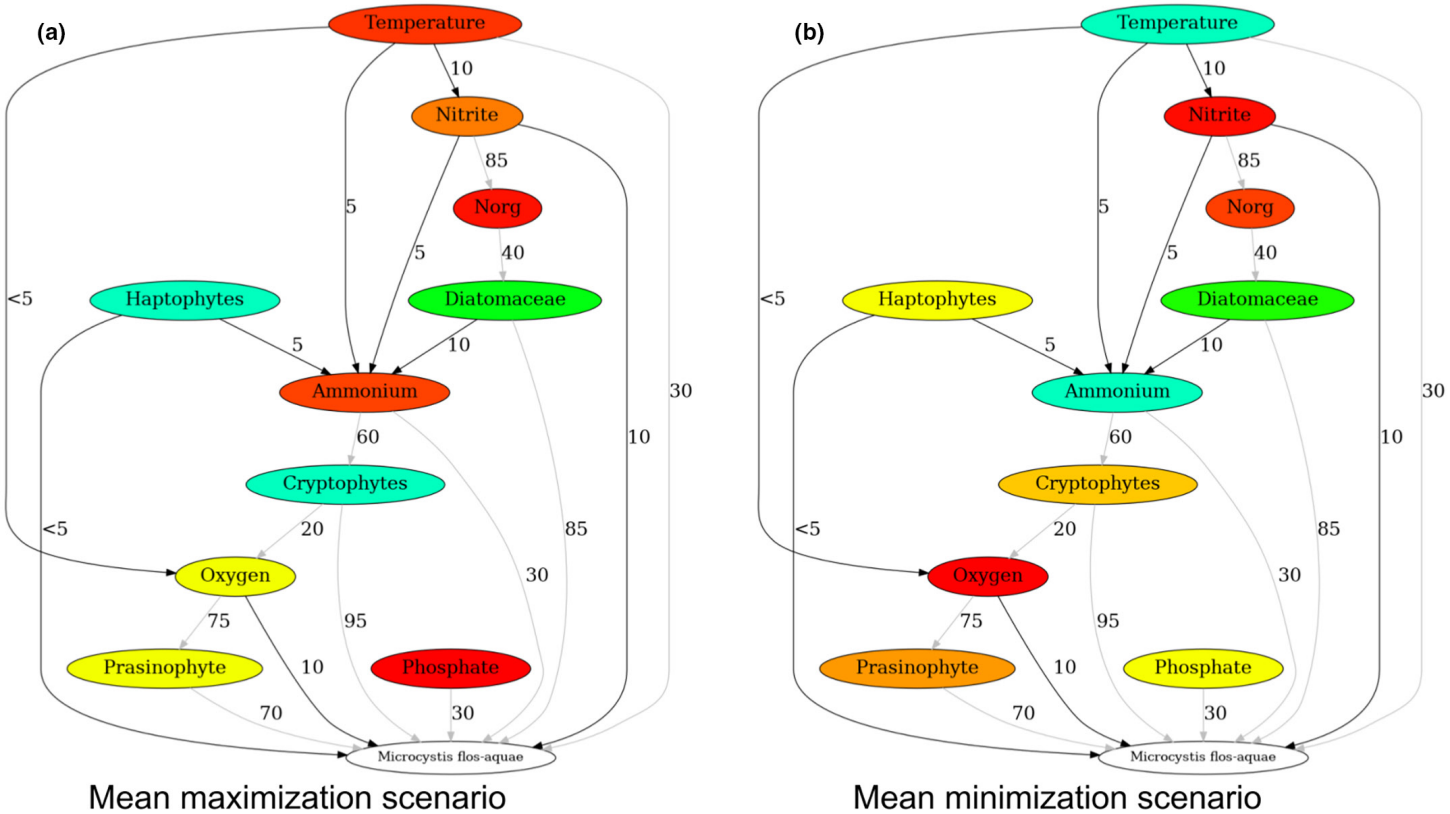
Permutations mean scenarios that: (a) maximize blooming probabilities (probability >.5, *n* = 87); and (b) minimize blooming probabilities (probability <.5, *n* = 34). (Cyan color indicates low mean value, red color indicates high mean value, e.g., red > orange > yellow > green > cyan). Numbers adjacent to the edges are the delayed effect in days. Black edges are of immediate effects (≤5 days), suggested as direct interactions; Gray edges are of long‐term effects (>5 days), suggested as indirect interactions. *M. flos‐aquae* biomass concentrations are categorized into two categories: low (below quantile 0.75) and high (quantile 0.75).

The sensitivity analysis (Figure 9) shows the mean contribution of the individual environmental parameters and the phytoplankton taxonomic groups to the model's output. The taxonomic groups present higher contribution to the model's output compared to the influence of the individual environmental variables. Mean scenarios were calculated based on permutations that produced high or low probabilities of *M. flos‐aquae* bloom formation (Figure 10). Lower probabilities of *M. flos‐aquae* blooms were associated with higher values of oxygen, prasinophytes, cryptophytes, and haptophytes but lower values of temperature, phosphate, and ammonium. In contrast, higher blooming probabilities were associated with higher temperature, phosphate, and ammonium values but lower oxygen, prasinophytes, cryptophytes, and haptophytes (mainly *Erkenia subaequiciliata*). Although the model is sensitive to nitrite, minor differences were observed in the mean scenario, probably due to non‐linear interactions of *M. flos‐aquae* and nitrite through its interactions with other phytoplankton and environmental parameters. These differences may pinpoint the potential factors favorable for developing higher *M. flos‐aquae* biomass.

A notable finding from our study was the measured lag (~30D) between changes in temperature and the subsequent effects on *M. flos‐aquae*. This temporal relationship provides compelling evidence that winter temperatures in Lake Kinneret are causal to the occurrence of spring blooms. Furthermore, we observed temperature differences when comparing the mean maximization scenario (Figure 10a) to the mean minimization scenario (Figure 10b). The mean maximization scenario depicted higher temperatures than the mean minimization scenario. Indeed, mean February temperatures of the upper 10 m are higher (17.3°C) in years of *M. flos‐aquae* blooms (2010–2015) compared to non‐bloom years (2004–2009) (16.5°C). This scenario‐based analysis provides valuable insights into the potential consequences of elevated temperatures on the ecological dynamics of Lake Kinneret. It suggests that under warmer conditions, there may be an increase in the intensity or frequency of algal blooms. In addition, the de‐stratification of the lake, following lake overturn, which typically occurs in the second half of December or in January, elevates high concentrations of nutrients from the lower anoxic nutrient‐rich layers of the lake to the nutrient‐poor upper layer. Mixing occurs 1–2 months before the *M. flos‐aquae* biomass peak. Mean scenarios also show the importance of higher ammonium and phosphate for *M. flos‐aquae* growth, suggesting that the overturn‐caused nutrient (ammonium and phosphate) supply to the upper productive layer is an important precondition for *M. flos‐aquae* bloom development.

**FIGURE 11:**
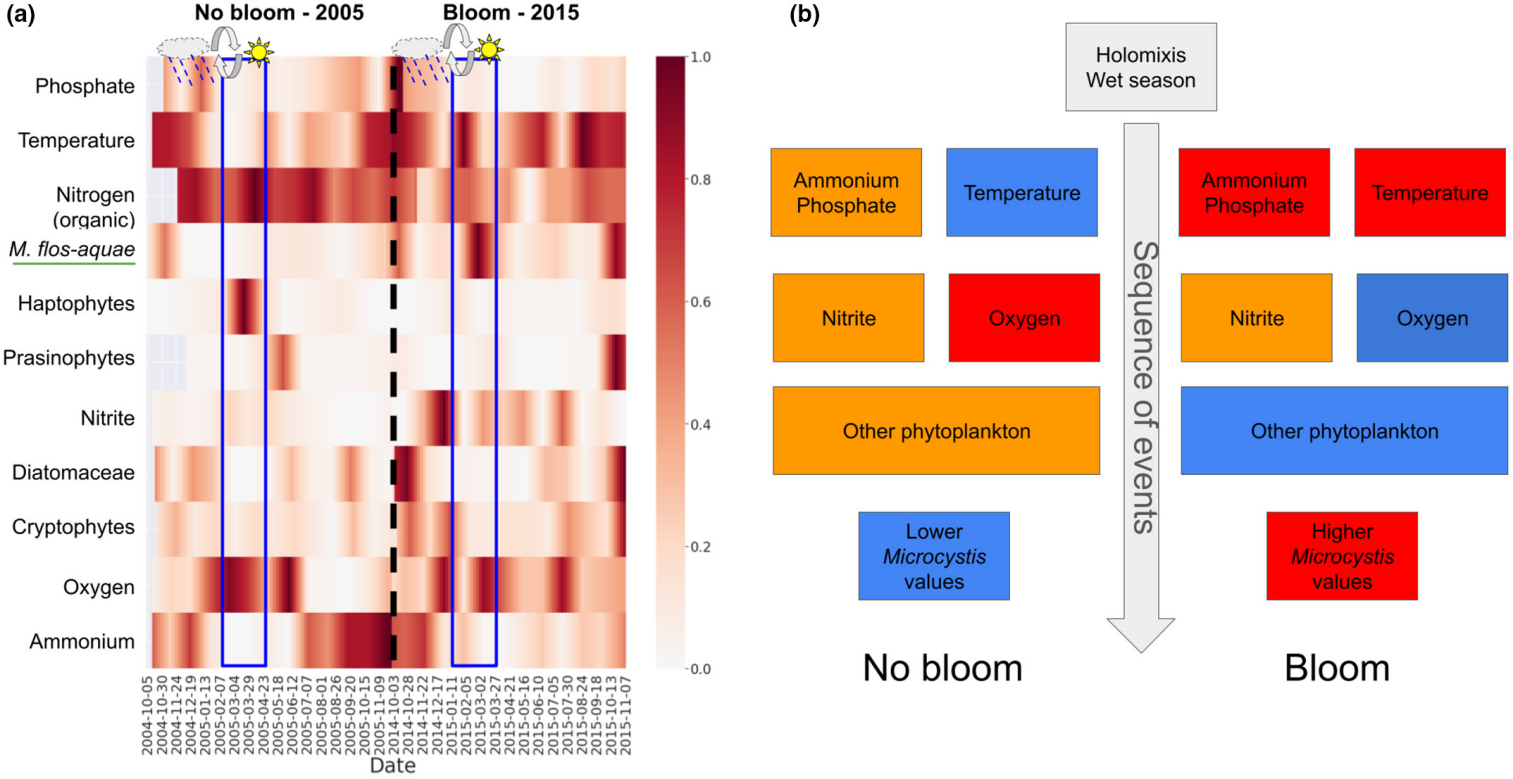
Demonstration of the preceding conditions that were suggested by the model in two periods: 2015—*Microcystis flos‐aquae* bloomed, and 2005—*M. flos‐aquae* did not bloom. (a) A comparison of the key environmental factors in the years 2005 and 2015. The values are normalized. The time series of the different variables of each year are shifted according to ECCM lag values relative to *M. flos‐aquae*; the color corresponds to the value: white color is 0, dark red is 1; blue rectangles mark the months of excepted *M. flos‐aquae* bloom (March); Event icons: rainy clouds mark the time of rainfalls, circular arrows mark the time of holomixis, and sun marks the time of increased temperatures followed the spring onset. (b) Schematic illustration that describes the sequence of events leading to *M. flos‐aquae* bloom, or not. Blue color is low values, orange color is mid values, and red color is high values.

We demonstrated how the preceding conditions that were suggested by the model are represented in two periods: when *M. flos‐aquae* bloomed (2015), and when it did not (2005) (Figure 11). Figure 11a presents the comparison of the key environmental factors that were identified by ECCM between these 2 years. This comparison demonstrates how real‐world environmental conditions affected *M. flos‐aquae* bloom formation. For instance, the bloom year (2015) coincided with higher temperatures, elevated phosphate and ammonium levels, and a lower abundance of competitor phytoplankton species. Conversely, the non‐bloom year (2005) is characterized by lower temperatures, reduced nutrient levels, and a higher abundance of competitor species.

## 4 DISCUSSION

Our framework for causal analysis represents an improvement over previous computational approaches in understanding the causes of cyanoHABs in Lake Kinneret. Traditional methods often relied on correlation‐based analyses, which only provided limited insights into the complex interactions and causal relationships among different factors (Rousso et al., 2020). These approaches do not consider all three essential aspects—delayed effects, causality, and event probabilities. In contrast, our framework utilizes a targeted and focused approach by constructing CCM–ECCM causal networks and developing BN models based on these networks. This allows a more comprehensive understanding of the underlying mechanisms driving bloom formation. Our framework considers the temporal aspect by considering the period leading up to the blooming events. This temporal perspective provides a better understanding of various factors' delayed effects and cumulative influences, leading to an explainable predictive capability.

We validated the CCM–ECCM approach using a synthetic time series studied previously by Ye et al. (2015). Moreover, we reconstructed a more challenging system in which the causal relations of the above system were weakened during the simulation. Although the sliding window approach performed better than the single frame CCM calculations, it missed two indirect interactions. This approach may only partially identify indirect interactions of longer delayed effects. The targeted CCM–ECCM approach performs better than the structure learning approach, which failed to identify the interactions. The model has revealed several key relationships among the factors influencing cyanoHABs formation in Lake Kinneret. These interactions shed some light on the complex interplay between variables and provide a deeper understanding of the underlying mechanisms. Some of the noteworthy interactions include the following.

### 4.1 Effect of temperature on *Microcystis flos‐aquae*

Higher temperatures may affect increased *M. flos‐aquae* growth (Figure 10), although the optimal temperature is varied between the different *Microcystis* species. Higher temperatures were found to promote the development of toxic sub‐populations (Davis et al., 2009; Imai et al., 2008). Another study in which the interactions of BN were constructed by domain experts (Moe et al., 2016) shows that phosphorus and temperature are important for the development of cyanobacterial blooms. The monthly mean in Lake Kinneret (Figure 3b) shows the highest values of *M. flos‐aquae* biomass in March, and relatively low values when the water gets warmest around June–August. It is important to note that the studies mentioned above were conducted based on lakes in Europe and North America, where *Microcystis* blooms occur in the summer, while in Lake Kinneret these blooms occur in the early spring.

### 4.2 Effect of nitrite and ammonium on *Microcystis flos‐aquae*

Ammonium, a reduced species of nitrogen, was found to be preferred by *M. flos‐aquae* over nitrate (Harke et al., 2016), while high nitrate levels were found to promote development of toxic *Microcystis* populations (Yoshida et al., 2007). As part of the nitrification process, nitrite is oxidized into nitrate in the presence of dissolved oxygen, which occurs in the deep part of Lake Kinneret between January and April following the annual overturn. Here, nitrite was found to be related also to lower values of *M. flos‐aquae*, suggesting that faster or earlier nitrification might promote its growth. It might also reflect *M. flos‐aquae* advantage by consuming ammonium over nitrite.

### 4.3 Effect of phosphate on *Microcystis flos‐aquae*

Higher phosphorus values cause increased *Microcystis* growth (Davis et al., 2009), although biomass increase of various *Microcystis* species may be favored by different phosphorus levels (Yue et al., 2014). According to our results, an increase in phosphate may affect *M. flos‐aquae* biomass. Kim et al. (2017) demonstrated that while the early growth stage of the Microcystis population is influenced by the nitrate‐to‐ammonium ratio and phosphate concentration, the maximum growth rate is primarily governed by the availability of a minimal phosphate concentration.

### 4.4 Effect of oxygen on *Microcystis flos‐aquae*

*Microcystis* is highly tolerant to low oxygen levels (Brunberg, 2002; Chen et al., 2018). The results show a relatively immediate influence of oxygen on *M. flos‐aquae*. Our approach successfully captured these relations (Figures 5a and 10), and suggests that lower oxygen levels, in the right conditions, promote *M. flos‐aquae* growth. This might be following the lack of other species blooms, which would increase oxygen levels due to photosynthesis. It also should be taken into account that a decrease in oxygen is associated with higher temperatures due to lower oxygen dissolution.

### 4.5 Effect of temperature on ammonium

Higher temperatures can increase the rate of decomposition of organic matter and as a result, increase ammonium levels in the water. Higher temperatures increase ammonia, nitrate, total nitrogen, and phosphate concentrations in freshwater ecosystems (Li et al., 2013). On the other hand, the main sources of nutrients for the *Microcystis* blooms are the increased winter inflows from watersheds and turnover‐driven destratification, which are annual processes in the monomictic Lake Kinneret, occurring during the coldest season and affecting nutrients level in the water body. In addition, following the turnover, large amounts of nitrite are oxidized to nitrate when nitrite arrives at the upper layers of the water column.

### 4.6 Effect of temperature on oxygen

As lake temperatures increase, the amount of dissolved oxygen it can hold decreases (Jankowski et al., 2006). Higher temperatures also increase phytoplankton photosynthesis rate, which in turn increases dissolved oxygen levels in the upper layers (Antonopoulos & Gianniou, 2003). The results of such dynamics might be a balancing act in Lake Kinneret, although this has not been confirmed.

### 4.7 Inter‐species interactions

The formation of *Microcystis* colonies was found to be related to its bacterial microbiome (Hoke et al., 2021; Wang et al., 2016) and the presence of multiple phytoplankton taxonomic groups (Zhang et al., 2019). The interactions between *Microcystis* and other community members are dynamic and bi‐directional (Omidi et al., 2021; Schweitzer‐Natan et al., 2023). According to our results, *M. flos‐aquae* is affected by other community members too. Inter‐species interactions in the lake might be due to mutualism, amensalism, or competition. Both amensalism and competition may reduce *Microcystis* growth rate. Competition delayed effect lag might be longer, while amensalism through allelopathy is assumed to follow a shorter lag time. Indeed, the analysis of CCM scores, delayed effect lag time, and model sensitivity results shed some light on the complex interactions of *M. flos‐aquae* with the phytoplankton community in its environment. The interactions of *M. flos‐aquae* with other phytoplankton species in the lake are stronger than their interactions with the environmental parameters (Figures 6a and 9). Even though *M. flos‐aquae* reacts faster to changes in environmental parameters (Figure 6b). These results are aligned with Chang et al. (2022) showing that in diverse ecosystems biodiversity effects are more important than environmental effects as drivers of biomass.

It is important to note that these identified interactions are based on the specific context of Lake Kinneret.

The lags between cause and effect, calculated by ECCM (Figure 10, Table S2), support the above evidence. The effect of temperature on oxygen, nitrite, and ammonium is immediate (1–2 weeks) in ecological time scales. The interactions between most of the biological variables and *M. flos‐aquae* are more prolonged (10–14 weeks), probably indirect, and occur due to inter‐species competition. In general, long delayed effects are either slow processes or indirect effects through the sequence of events.

Intuitively, the importance of higher ammonium and phosphate for *M. flos‐aquae* growth is in contrast to the temperature because with overturn, the temperature of the upper layer decreases. This may lead to shallow warm temperatures while most of the water column is cooler. However, *Microcystis* blooms occur only following sufficiently warm, calm winter days (Hozumi et al., 2020). Here we use temporal information of the lag between the cause and the effect (Figures 10 and 11), which shows that multiple processes of different time ranges affect *M. flos‐aquae* blooms. First, during the winter, around November–February, there are increased loads of nitrite, ammonium, and phosphate from watershed into the lake. During this time, the turnover occurs, which mixes the water, increasing nutrient levels in the upper layer of the lake. Later, warmer temperatures during the winter and early spring affect oxygen and nutrient levels, which in turn affect the phytoplankton of the different taxonomic groups. Global warming was found to delay the overturn in lakes (Anderson et al., 2021), which reduces nitrite levels in the upper layer. In the absence of other blooming species in the lake, this sequence of events increases the probability of *M. flos‐aquae* blooms and was demonstrated using two distinct periods (2015, bloom; 2005, no bloom) in Lake Kinneret (Figure 11).

Synergistic effects refer to the phenomenon where the joint influence of multiple variables on an outcome is greater than the sum of their individual effects. This effect is particularly relevant when studying complex systems, where the interactions between variables can lead to non‐linear dynamics. In BNs, the network structure, represented as a DAG, allows for identifying synergistic and cumulative interactions among variables (Simeoni et al., 2023). Indeed, sensitivity analysis of the BN model presents a relatively weak effect of each parameter (Figure 9). However, their synergistic effect on the output values (*M. flos‐aquae* blooming probability) is of a broader range (Figure 8). For example, higher temperatures in January–February are not enough to induce a bloom in the spring. Inferring this condition using the BN model shows that the probability of having *M. flos‐aquae* bloom when temperature levels are higher is .47. Lower oxygen levels and lower competing phytoplankton values support the impact of temperature (probability increase to .5), while the presence of sufficient ammonium or nitrite increases the probability to >.89. This might be explained by considering a synergistic effect between environmental and biological parameters as demonstrated above. The results could indicate that while biological parameters are individually important and exhibit strong statistical associations, their effects might be enhanced or modulated by the presence of certain environmental conditions. The combination of both sets of parameters working together may result in a more comprehensive understanding of the system's behavior, with the environmental parameters playing a crucial role in shaping and driving the overall dynamics.

The proposed framework presents a novel approach to understanding complex processes in ecological systems. However, there are still specific weaknesses that should be acknowledged: (a) Uncertainty in BN modeling: the accuracy of the BN models heavily relies on the availability and quality of data for training and validation. In addition, thresholds used for categorization and insufficient or noisy data may affect the reliability and generalizability of the models. (b) Data limitations: The framework's effectiveness is contingent on comprehensive and high‐quality data availability. Incomplete or sparse data may limit the ability to accurately identify and capture all relevant causal relationships. (c) Simplified representation: while the framework provides a more comprehensive understanding of the underlying mechanisms, it still relies on simplifications and assumptions to model the complex interactions among variables. The relationships between the different variables were simplified due to data categorization because the categorized dataset is a simplified representation of the data. This simplification may overlook specific interactions within the system, potentially leading to incomplete conclusions. In addition, BN model assumes that feedback loops are absent. (d) Hidden variables: not directly observed but impact the observed data. Incorporating hidden variables would allow for a more comprehensive representation of the causal structure. (e) The ECCM results still depend on subjective human interpretations. The pattern produced by the ECCM analysis is not always ideal, for example, consisting of a single and sharp peak for each of “x → y” and “y → x”. For instance, “x → y” might present a major peak at some lag *n* < 0, but also a clear shoulder or even a lower peak between lag 0 to some other value >*n* and <0. In this case x affects y relatively immediately, but at lag *n* there is a greater mutual information. Should the lower peak which is closer to 0 be selected, or the highest peak? Here we selected the highest peak at lag <0. And (f) Interactions involving biological components tend to be masked by long‐term trends, which should be considered.

Despite these weaknesses, the framework can offer valuable insights and contribute to understanding ecological systems. It provides a targeted and focused approach that considers the causal interactions and the temporal aspect, allowing for a better understanding of lagged effects and cumulative influences. Incorporating causality and dependent probabilities provides the framework with a more explainable predictive capability than traditional analyses.

Further improvements can be made in data collection and model refinement to address the weaknesses. Gathering more comprehensive and high‐quality data, including long‐term and continuous monitoring, can enhance the accuracy and robustness of the CCM causal networks and BN models. Additionally, incorporating unobserved variables can capture a more comprehensive representation of complex ecological systems.

We presented a computational framework of coupled CCM–ECCM and BN for causal analysis of complex ecosystems. As a case study, we focused on the bloom‐forming *M. flos‐aquae* species in the subtropical Lake Kinneret. Given the causal interactions identified by CCM–ECCM and reviewed by domain experts, the structure of the causal network can be used as a basis for a BN model. Although BN has been used in an ecological context, this is the first attempt to use BN models in conjunction with CCM and ECCM. Here, this conjunction was made to understand the freshwater ecosystem.

*Microcystis flos‐aquae* in Lake Kinneret is associated, by complex interactions, with the phytoplankton community but also driven by environmental variables such as temperature, ammonium, nitrite, and phosphate.

## AUTHOR CONTRIBUTIONS

**O. Tal:** Conceptualization (lead); data curation (lead); formal analysis (lead); investigation (lead); methodology (lead); resources (lead); software (lead); supervision (lead); validation (lead); visualization (lead); writing – original draft (lead); writing – review and editing (equal). **I. Ostrovsky:** Conceptualization (supporting); formal analysis (supporting); methodology (supporting); writing – review and editing (supporting). **G. Gal:** Conceptualization (supporting); formal analysis (supporting); methodology (supporting); writing – review and editing (equal).

## CONFLICT OF INTEREST STATEMENT

The authors have no conflicts of interest to declare.

## Supporting information


Appendix S1.

## Data Availability

The code of this study is available on GitHub at https://github.com/ot483/ecol_evol_2023. The data used in this study have been deposited in the Zenodo repository and are publicly available and can be accessed and downloaded from the Zenodo [https://zenodo.org] under the corresponding DOI [10.5281/zenodo.11207098].
