# Supplementary material for: A framework for identifying factors controlling cyanobacterium Microcystis flos‐aquae blooms by coupled CCM–ECCM Bayesian networks
Source: Ecol Evol. 2024 Jun 25;14(6):e11475. doi: 10.1002/ece3.11475 (PMC11199127; doi:10.1002/ece3.11475)
Supplement: Supplementary file 1 — Appendix S1. [file ECE3-14-e11475-s001.docx]

**Supplementary – Figures and Tables**


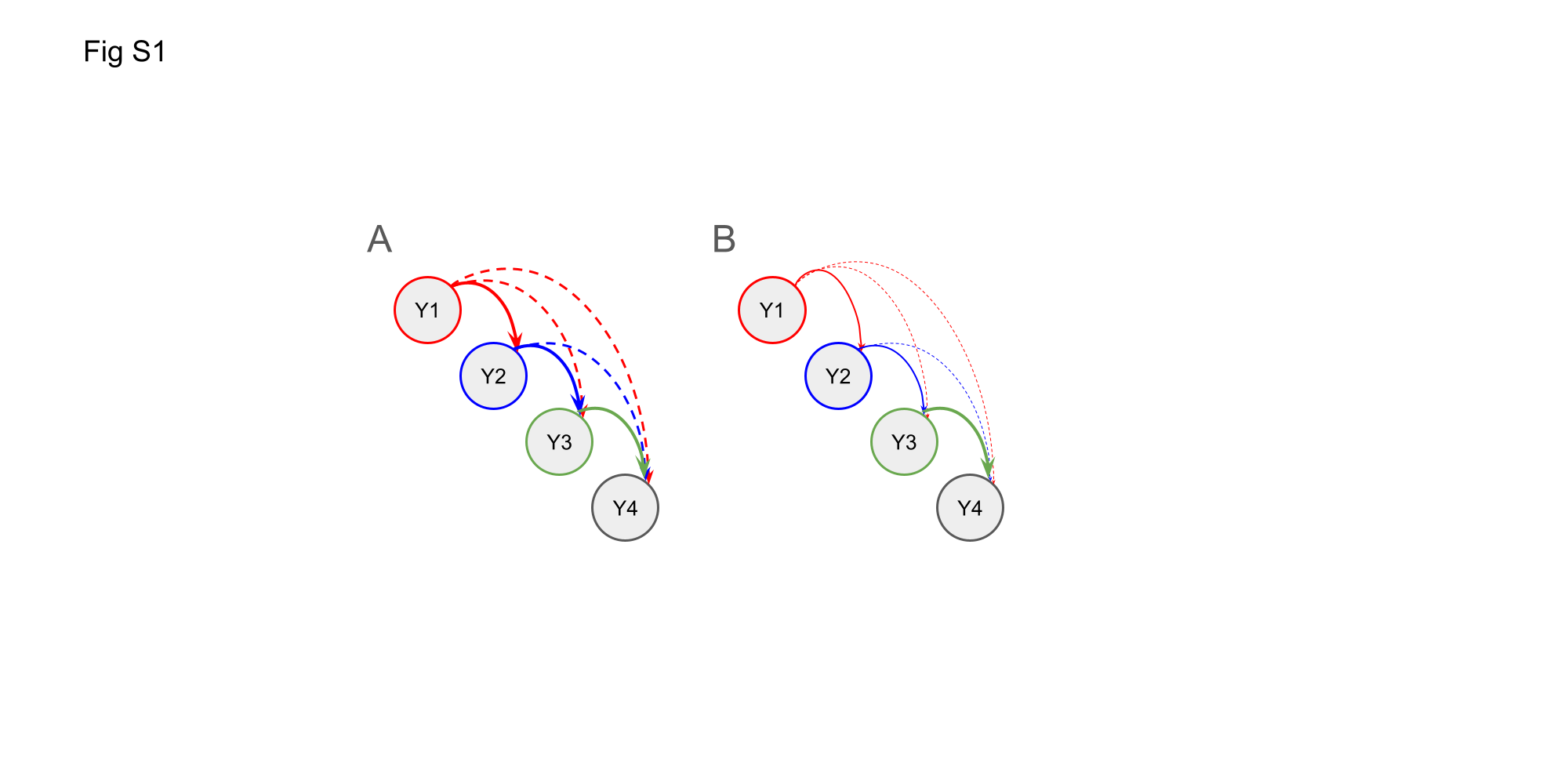


Figure S1 – Schematic illustration of the simulated systems described by Sugihara et al (2015), and was used to test the proposed framework. The original system is composed of four components: y1 → y2 → y3 → y4, and consists direct and indirect interactions. Red – is the interactions of y1; blue – is the interactions of y2; green – is the interactions of y3; The width of the arrows is proportional to interaction strength; solid arrow indicates a direct interaction; dashed arrow indicates an indirect interaction; Two systems were used: (A) homogeneous, similar to the system described by Sugihara. (B) heterogeneous, in which the direct interactions of y1 → y2 and y2 → y3 were weakened, therefore also weakened the indirect interactions of y1 → y3, y1 → y4 and y2 → y4.


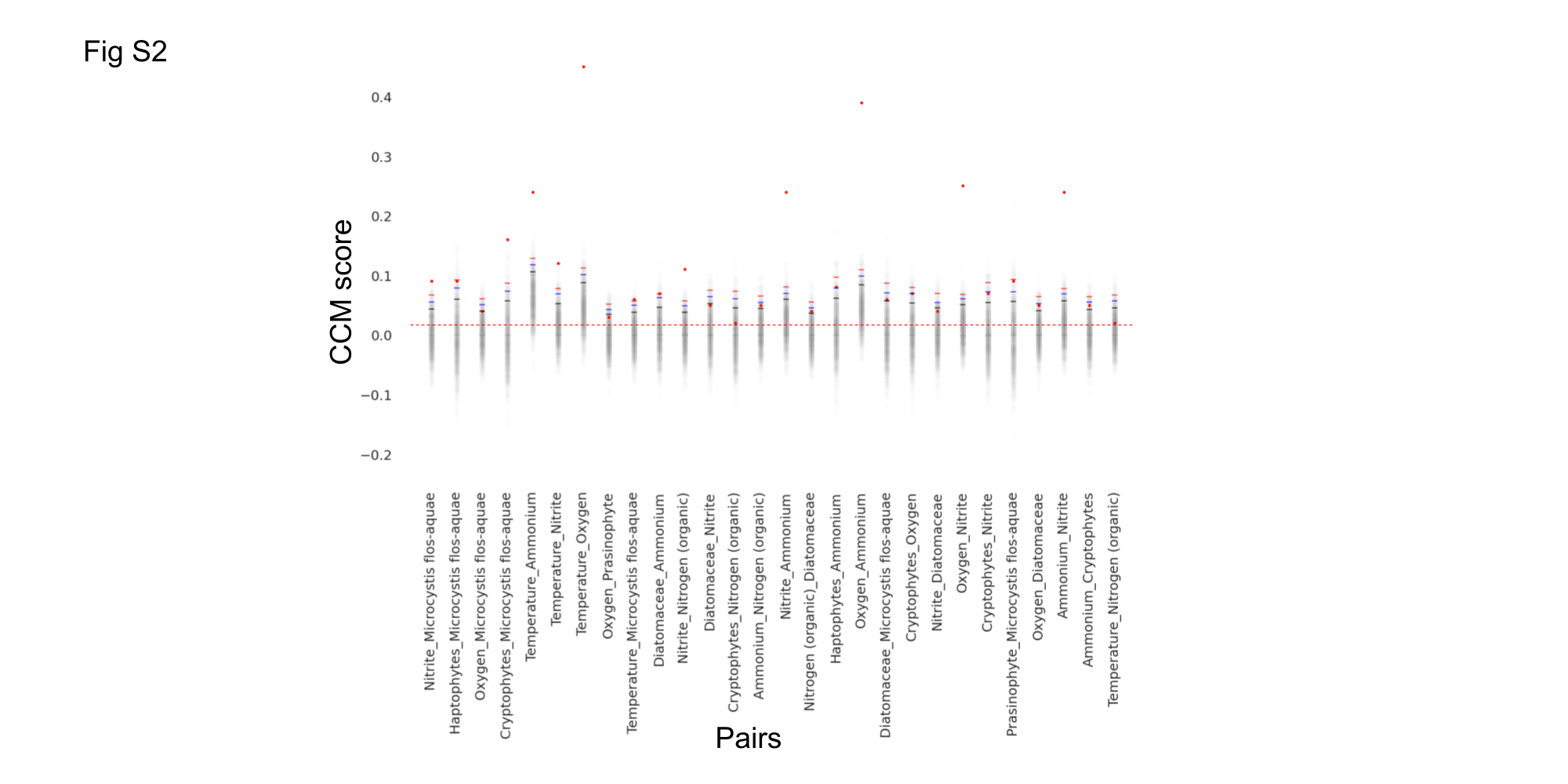


**Figure S2 - Distribution of CCM values from 1,000 surrogate time series compared to observed CCM values for all pairwise relationships between variables. The gray densities represent the distribution of CCM values from 1,000 surrogate time series for each pair. The horizontal lines within each distribution represent the 0.9 (lower, black), 0.95 (middle, blue), and 0.975 (upper, red) quantiles of the surrogate CCM distributions. The red dots represent the observed (true) CCM values calculated from the original time series. The true values (red dots) are considered significant if they are higher or equal to the 0.9 quantile score.**


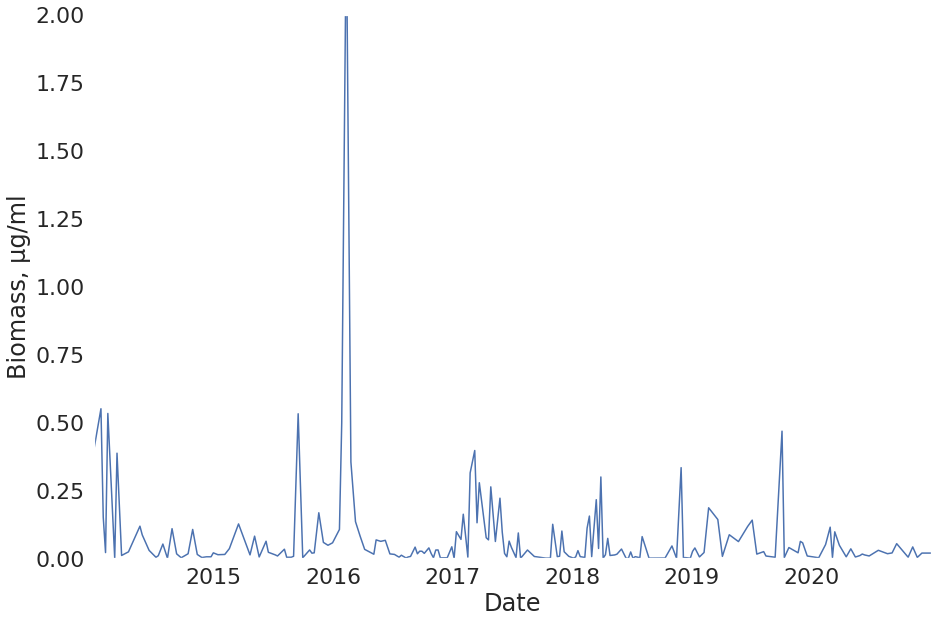


Figure S3 – *M. flos-aquae* values between years 2014-2020, weekly, linear interpolation of missing values.


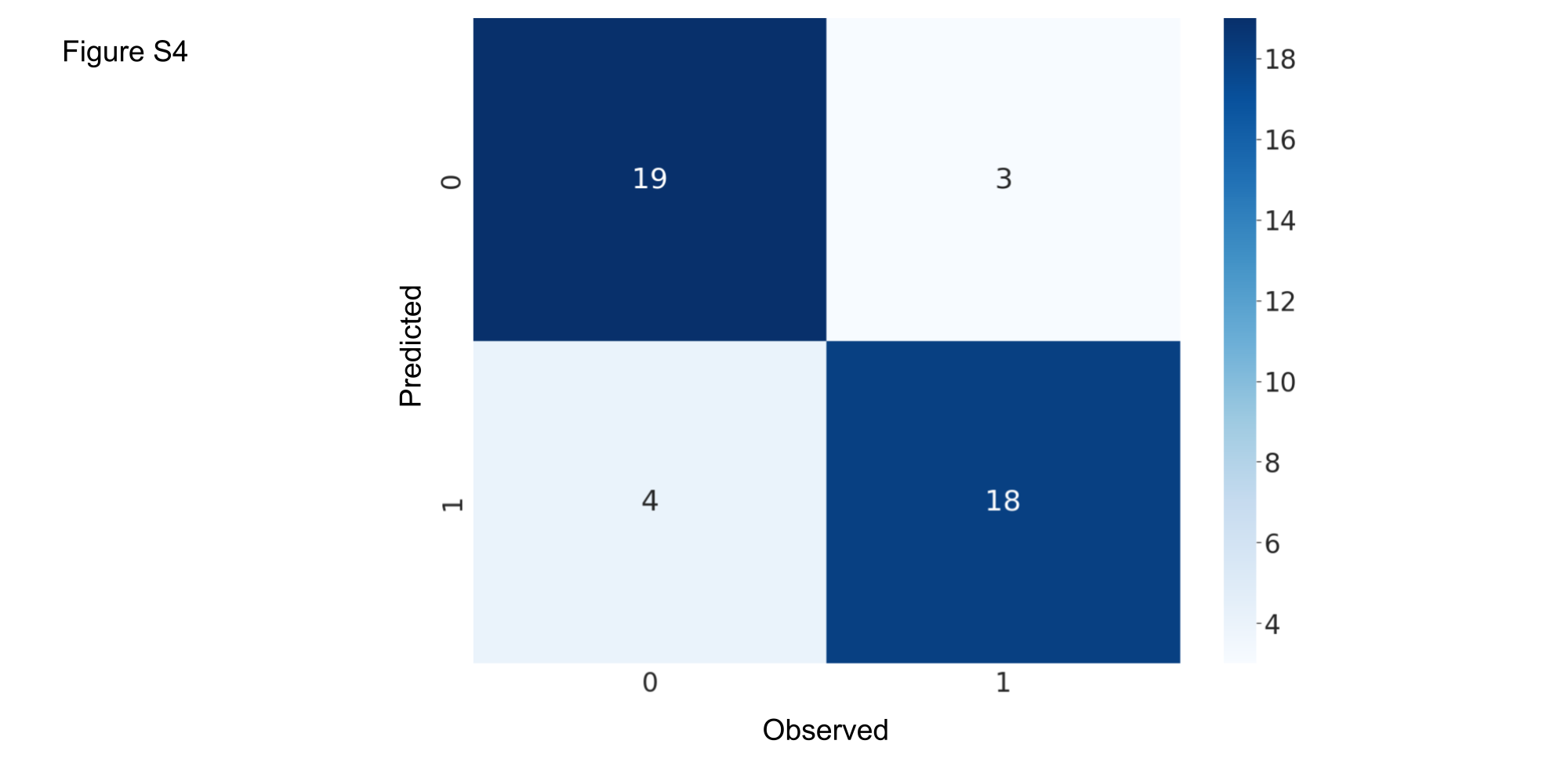


|  |  |  |
| --- | --- | --- |

**Figure S4 - Confusion matrix showing the performance of the Bayesian Network (BN) model on the test set. The x-axis represents the observed class, and the y-axis represents the predicted class. The diagonal cells (shaded in blue) show the number of correct predictions. The overall accuracy of the model is 84.1%.**

**Table S1 - Categorization thresholds for water quality variables.**

**The table describes the thresholds used to categorize the variables used in this study into three categories. The units used for each variable (mg/l, NTU, etc.) are under the Unit columns. Description of the categorization scheme is under the Categories and cutoffs column. Variable names are under the Variable column. The categories are assigned numerical labels (0, 1, 2). For pH, and temperature specific ranges are used for categorization. For *M. flos-aquae* and phytoplankton taxa (Prasinophyte, Chlorophyta, etc.), a single quantile is used to create a binary categorization (0/1).**

| **Variable** | **Unit** | **Categories and cutoffs** |
| --- | --- | --- |
| Nitrite | mg/l | quantile 0.55/quantile 0.85, '0'/'1'/'2' |
| Nitrate | mg/l | quantile 0.55/quantile 0.85, '0'/'1'/'2' |
| Ammonium | mg/l | quantile 0.55/quantile 0.85, '0'/'1'/'2' |
| Oxygen | mg/l | quantile 0.55/quantile 0.8, '0'/'1'/'2' |
| Organic nitrogen | mg/l | quantile 0.3/quantile 0.75, '0'/'1'/'2' |
| Phosphate | mg/l | quantile 0.5/quantile 0.85, '0'/'1'/'2' |
| Prasinophyte | Biomass, μg/ml | quantile 0.75, '0'/'1' |
| Diatomaceae | Biomass, μg/ml | quantile 0.75, '0'/'1' |
| Haptophytes | Biomass, μg/ml | quantile 0.75, '0'/'1' |
| Cryptophytes | Biomass, μg/ml | quantile 0.75, '0'/'1' |
| Temperature | Celsius | 18.5/21.5, '0'/'1'/'2' |
| *Microcystis flos-aquae* | Biomass, μg/ml | quantile 0.75, '0'/'1' |

|  |  |  |  |
| --- | --- | --- | --- |

**Table S2 - CCM scores and ECCM delayed effects. The table shows the causal variable (X1) and the affected variable (X2) for each calculated CCM prediction skill (*p*). The prediction skill is a value between 0 and 1 that quantifies the strength of the relationship between the causal and affected variables. Higher values indicate a stronger association. The delayed effect (time steps) indicates the number of time steps it takes for the causal variable to affect the affected variable. A value of 0 means there is no delay (instantaneous effect).**

| **X1 (Causal variable)** | **X2 (Affected variable)** | **Prediction skill (*p*)** | **Delayed effect (time steps)** |
| --- | --- | --- | --- |
| Temperature | *Microcystis flos-aquae* | 0.06 | 6 |
| Temperature | Nitrite | 0.12 | 2 |
| Temperature | N (organic) | 0.02 | 10 |
| Temperature | Oxygen | 0.45 | 0 |
| Temperature | Ammonium | 0.24 | 1 |
| Diatomaceae | Nitrite | 0.05 | 1 |
| Diatomaceae | Ammonium | 0.07 | 2 |
| Nitrite | Diatomaceae | 0.04 | 2 |
| Nitrite | *Microcystis flos-aquae* | 0.09 | 2 |
| Nitrite | N (organic) | 0.11 | 17 |
| Nitrite | Ammonium | 0.24 | 1 |
| N (organic) | Diatomaceae | 0.04 | 8 |
| Haptophytes | Ammonium | 0.08 | 1 |
| Oxygen | Diatomaceae | 0.05 | 10 |
| Oxygen | Prasinophyte | 0.03 | 15 |
| Oxygen | *Microcystis flos-aquae* | 0.04 | 2 |
| Oxygen | Nitrite | 0.25 | 2 |
| Oxygen | Ammonium | 0.39 | 3 |
| Cryptophytes | Nitrite | 0.07 | 2 |
| Cryptophytes | N (organic) | 0.02 | 10 |
| Cryptophytes | Oxygen | 0.07 | 4 |
| Ammonium | Nitrite | 0.24 | 0 |
| Ammonium | N (organic) | 0.05 | 0 |
| Ammonium | Cryptophytes | 0.05 | 12 |
| Diatomaceae | *Microcystis flos-aquae* | 0.06 | 17 |
| Prasinophyte | *Microcystis flos-aquae* | 0.09 | 14 |
| Haptophytes | *Microcystis flos-aquae* | 0.09 | 0 |

**Table S3 - Performance of BN models constructed using interactions exceeding different surrogate quantile thresholds. The table shows the performance of BN models constructed using interactions identified with different quantile levels. Three surrogate quantile thresholds (0.90, 0.95, and 0.975) were used to filter potential interactions. For each threshold: Quantile - The value of the surrogate quantile threshold used to filter interactions. Number of nodes in DAG - The number of variables included in the final DAG of the BN model. Number of interactions in DAG - The number of interactions identified between variables in the final DAG. Accuracy - The proportion of correctly classified instances in the test set. AUC - The area under the curve of the roc, a which is model's ability to discriminate between the classes.**

| **Quantile** | **Number of nodes in DAG** | **Number of interactions in DAG** | **Accuracy** | **AUC** |
| --- | --- | --- | --- | --- |
| 0.9 | 10 | 20 | 0.841 | 0.842 |
| 0.95 | 9 | 16 | 0.876 | 0.863 |
| 0.975 | 9 | 14 | 0.780 | 0.781 |
